# Reincarnations of the phase separation problem

**DOI:** 10.1038/s41467-020-20360-4

**Published:** 2021-02-10

**Authors:** Ruo-Yu Dong, Steve Granick

**Affiliations:** 1 Center for Soft and Living Matter, Institute for Basic Science (IBS), Ulsan, 44919 South Korea; 2 Departments of Chemistry and Physics, Ulsan National Institute of Science and Technology (UNIST), Ulsan, 44919 South Korea

**Keywords:** Engineering, Materials science, Applied physics

## Abstract

Phase separation is familiar and useful, yet opportunities to manipulate it are surprisingly subtle and complex.

Two just-published papers^1,2^ remind us that in approaching any new scientific problem, there are generally three stages of reaction. Our first impression is of difference and strangeness. But once superficial dissimilarities are pierced, our feeling becomes the opposite extreme. Everything now appears fundamentally the same. All that we can now see are the underlying commonalities, with different forms according to their settings. Only in the third stage does real knowledge begin. We look anew for differences, not this time for those obvious elements that hit the eyes of newcomers, but rather for subtleties whose importance structures the problem.

Phase separation is an evergreen subject that reinvents itself continually. Starting early on, scientists and engineers sought to control items such as steel microstructure and to understand workaday items such as why oil floats on soup, leading in the 19th and early 20th century to focus on nucleation and growth^3^. Then came a surprise—the identification of spinodal decomposition^4^; it was followed by an era of exploring the spinodal decomposition formalism in many manifestations, a pattern that to some extent continues today. Independently, explosive advances follow from progress in understanding critical points, an accomplishment recognized by a Nobel Prize in the latter part of the 20th century^5^. Surveying the ensuing scientific literature one finds that the phrase “phase separation” now is used progressively less often. During the 21st century, this loss is balanced by increasing the use of the phrase “self-assembly”. For experimentalists, much of the distinction between these concepts can be semantic. Both concepts, phase separation and self-assembly, share the concern with understanding how order appears from disorder.

A new coarsening mechanism in phase separation^1^ and bicontinuous nanoparticle gels^2^ are reported from the groups of Hajime Tanaka at the University of Tokyo and Yun Liu at the University of Delaware/National Institute of Standards and Technology, respectively. On first reading, the subject seems to differ from what professors teach students about phase separation—where are the phase diagrams, tie lines, and other standard thermodynamic quantities? These studies, dwelling instead on how processes evolve in time, seem alien to the textbook^3^ view. On the second reading, the phase-separated structures are familiar—coarsening^1^, a standard feature of nucleation and growth, and bicontinuous structures^2^, standard for spinodal decomposition, so from this perspective, readers can feel comfortable. It requires third reading to see where these interesting studies go beyond the standard view.

Both studies exemplify how a mere difference in mobility can influence even the long-time structural appearance of two separating components—one of them relatively fast, the other relatively slow. This problem doesn’t present itself in the usual textbooks which unspokenly presume similar mobility of the phase-separating components, with the result that this issue doesn’t even enter the statement of the problem^3^. Yet the paper by Tateno and Tanaka^1^ demonstrates unconventional *t*^1/2^ power-law growth of time (*t*)-dependent structure in liquid–liquid phase separation, shockingly faster than the *t*^1/3^ dependence that normally follows from conventional analysis for the Lifshitz–Slyozov–Wagner mechanism, Brownian coagulation mechanism, and even nonequilibrium thermodynamics when energy-dissipating systems are mapped onto a formalism of equivalent free energy^6^. The engineering-oriented paper by Xi et al. likewise demonstrates findings contrary to textbook expectations. Adding nanoparticles designed to be miscible in only one phase of a phase-separating system, the authors assert that their tiny particles avoid the interface because their small size and high charge avoid the traditional rule of thumb that particles and nanoparticles ubiquitously segregate to interfaces.^7^ Instead, the authors report that nanoparticles stabilize the phase-separated structure because they jam one of the liquid phases. If substantiated by direct test, this would provide an interesting extension to the traditional Pickering emulsion concept. Freezing bicontinuous structures into place using this strategy, Xi et al.^2^ argue that their process to accomplish this may be useful from technology and materials processing standpoints.

Phase separation modulated by mismatched time scales, a process conceived decades ago by Tanaka^8^, has come of age after a long childhood. Seeds planted by the early concepts^8,9^ are likewise sprouting in other laboratories. For example, the group of E. Dufresne at the ETH Zürich investigates Ostwald ripening, finding provocative differences from classical expectations when this happens within a polymer network such that droplet growth comes at the cost of elastic stiffening in the surrounding medium^10^. The toggled interactions proposed as a concept by the group of J. Swan at Massachusetts Institute of Technology^11^ could evidently also be extended to become an additional control variable. The concept of using nanoparticles to jam interfaces, thereby bypassing the conventional temperature or pressure quench, has been used in multiple systems by the group of T.P. Russell, with an impact that extends to the patent literature^12^. Independently in multiple laboratories, the view is gelling that beyond conventional thermodynamic variables, the time variable belongs as an axis on phase diagrams, not literally, but when one thinks about the problem conceptually.

Materials physicists and engineers previously were (of course) aware from experience that time matters. But this was regarded chiefly as a nuisance, something akin to trying to play sports during bad weather. These new papers show beautiful conceptual novelty^1^ and practical utility^2^. When one speculates how these ideas may spread in the future, the latter is especially notable when one considers the high value that the era in which we live gives to having technology impact. It is natural to anticipate further advances when, in further studies the time variable, according to which interactions are toggled, is made syncopated and enriched with rhythm and tempo, as outlined speculatively in Fig. 1.

**Fig. 1:**
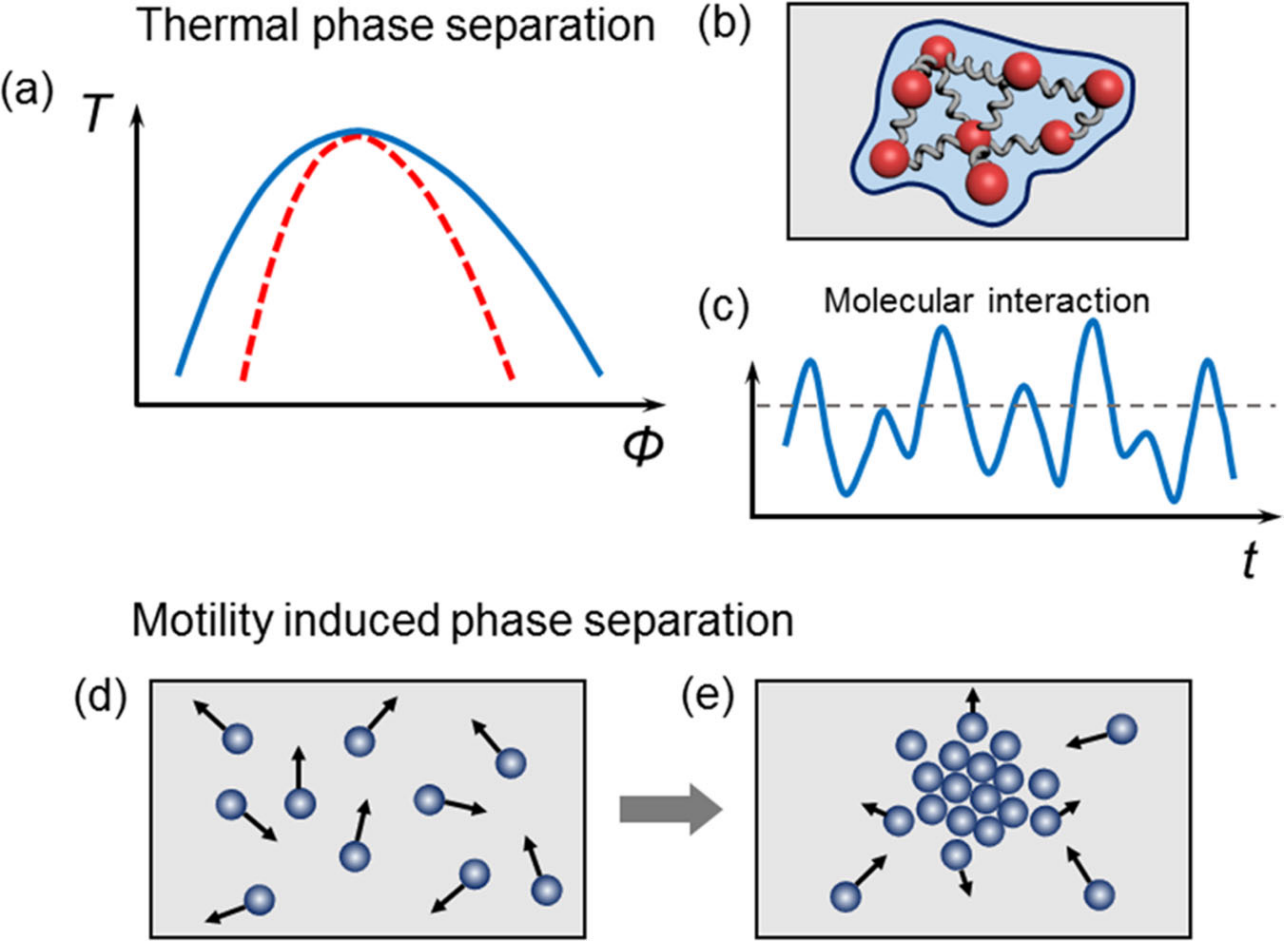
Deconstructing phase separation—old and new. **a** The traditional liquid-liquid phase diagram, sketched here with axes of temperature and volume fraction, is controlled by thermodynamic variables only. This concept led in the 19th and early 20th century to use strategies to control items such as materials microstructure, and to intellectual inquiry such as why oil floats on soup, all of this based on nucleation and growth corresponding to deep quenches below the binodal (solid blue) line^3^. Then came a surprise—the identification of spinodal decomposition (dotted red line)^4^. **b** In modern times, Tanaka and coworkers^1,6^ have demonstrated the novelty of thermal phase separation when the phase-separating components respond over drastically different response times, shown here schematically as viscoelasticity resulting from time-dependent elastic (red) interactions in the viscous (blue) liquid. **c** Other modern work toggles molecular interactions themselves during the course of phase separation, shown here schematically as modulation in time. **d** Out-of-equilibrium active matter systems present radically different situations. These active, motile particles have directional motion because they consume energy, shown here schematically as arrows on the moving particles. **e** States of motility-induced phase separation, controlled by differential motility of individual active particles, can result. The regions of high-density form because moving objects tend to accumulate where they move more slowly. Here the differential arrow lengths denote differential motility.

Out-of-equilibrium self-organization is interesting even more generally. Going even farther beyond the standard view of phase separation, the new field of active matter considers “phases” and “phase diagrams” under conditions where motility (“self-propulsion”), is the heart of the problem^13,14^. In this view, phase separation is induced by motility itself—not enthalpy and entropy. Moving objects, individually self-propelled by consumption of energy, tend to accumulate where they move more slowly. The resulting phase-separated structures often look visually like nucleated or spinodal structures known from thermal phase separation. At a deep level, that they are so similar to the casual eye is not coincidental—elegant theory shows that the situations can be mapped onto one another conceptually, so similar geometrical patterns of phase separation follow naturally from equations whose structure is similar^6^.

However, most current experiments in this field still struggle with the challenge of how to produce active matter systems with controllable mutual interactions in the laboratory^13^. Despite experimental tours de force, and provocative analogies to living systems of animals, fish, and bird, it remains easier to do so on the computer than in the laboratory^14^. The concept of effective temperature remains ambiguous^15^. Details of this correspondence are, at the time of this writing, too little explored experimentally. The search for practical applications is, at present, mostly limited; known examples include the construction of membrane-less organelles with possible biophysical action^16^, the speculative use of isolated active particles in medicine^17^, and the construction of robot swarms of various kinds^18^. Regarding these examples of applications, a survey of the experimental literature shows too little data about phase diagrams. The laboratory studies of phase separation in the active matter are mostly in two-dimensional systems, but experimental studies of thermal phase separation are usually three-dimensional. Experiments with active matter are (so far) geared toward understanding individual clusters, swarms, or organelles, not yet system-spanning such as bicontinuous structures that are standard to find in thermal phase separation, and not yet in the presence of viscoelastic^1^ or jammed^2^ background. To bridge the obvious gaps in this long list will be a significant agenda for the future.

Now in the second quintile of the 21st century, we are at a tipping point. The study of classical thermal phase separation is being enriched intellectually by relaxing the previously-strict injunction that thermodynamics and kinetics are distinct subjects. Independently, out-of-equilibrium systems draw massive attention. It is fascinating to observe the evolution of science in a microcosm. Each chapter of progress is accompanied by a new generation of young scientists. In the field of phase separation, as in so many others, science often advances one funeral at a time^19^.
